# Functional shift with maintained regenerative potential following portal vein ligation

**DOI:** 10.1038/s41598-017-18309-7

**Published:** 2017-12-22

**Authors:** Tibor Kovács, Domokos Máthé, András Fülöp, Katalin Jemnitz, Attila Bátai-Konczos, Zsuzsanna Veres, György Török, Dániel Sándor Veres, Ildikó Horváth, Krisztián Szigeti, László Homolya, Attila Szijártó

**Affiliations:** 1 Hepato-Pancreatico-Biliary Surgery Research Center Hungary, 1st Department of Surgery, Semmelweis University, Budapest, Hungary; 2 Department of Biophysics and Radiation Biology, Semmelweis University, Budapest, Hungary; 3 CROmed Translational Research Centers, Budapest, Hungary; 4 Institute of Organic Chemistry, Research Centre for Natural Sciences, Hungarian Academy of Sciences, Budapest, Hungary; 5 Institute of Enzymology, Research Centre for Natural Sciences, Hungarian Academy of Sciences, Budapest, Hungary

## Abstract

Selective portal vein ligation (PVL) allows the two-stage surgical resection of primarily unresectable liver tumours by generating the atrophy and hypertrophy of portally ligated (LL) and non-ligated lobes (NLL), respectively. To evaluate critically important underlying functional alterations, present study characterised *in vitro* and *vivo* liver function in male Wistar rats (n = 106; 210–250 g) before, and 24/48/72/168/336 h after PVL. Lobe weights and volumes by magnetic resonance imaging confirmed the atrophy-hypertrophy complex. Proper expression and localization of key liver transporters (Ntcp, Bsep) and tight junction protein ZO-1 in isolated hepatocytes demonstrated constantly present viable and well-polarised cells in both lobes. *In vitro* taurocholate and bilirubin transport, as well as *in vivo* immunohistochemical Ntcp and Mrp2 expressions were bilaterally temporarily diminished, whereas LL and NLL structural acinar changes were divergent. *In vivo* bile and bilirubin-glucuronide excretion mirrored macroscopic changes, whereas serum bilirubin levels remained unaffected. *In vivo* functional imaging (indocyanine-green clearance test; ^99m^Tc-mebrofenin hepatobiliary scintigraphy; confocal laser endomicroscopy) indicated transitionally reduced global liver uptake and -excretion. While LL functional involution was permanent, NLL uptake and excretory functions recovered excessively. Following PVL, functioning cells remain even in LL. Despite extensive bilateral morpho-functional changes, NLL functional increment restores temporary declined transport functions, emphasising liver functional assessment.

## Introduction

With a worldwide ranking of sixth in incidence and third in cancer-related deaths, liver malignancies represent a chief challenge for liver surgery^1^. Doubtlessly, the sole curative treatment is complete tumour elimination, preferably by surgical resection^2,3^. Meanwhile, much different clinical management is required in patients with large liver tumours, requiring extensive resections, sparing only an undersized ‘future liver remnant’ (FLR), and thereby risking the development of liver failure. In such cases, the ‘resection of unresectable’ may still be realised in two stages and an intercalated waiting period through preoperative FLR enlargement protocols^2^. A key solution for overcoming this problem is portal vein occlusion (PVO) procedures^4,5^. PVO is performed by either the ligation (PVL) or embolization (PVE) of selected portal vein tributaries, leading to the atrophy of portally deprived lobes and the parallel compensatory hypertrophy of portally perfused lobes; a special form of liver regeneration (LR), the latter being nature’s enigmatic phenomenon visionarily foretold by the Promethean legend^6–8^. LR has thoroughly been investigated by a number of studies utilising partial hepatectomy (PH)^9^. Whilst physiological drives and features of PVO- and PH-induced LR are matching, PVO has to be regarded as a delicately unique form of LR. Firstly, two directly opposite adaptive physiological processes (atrophy-hypertrophy complex) are maintained strictly separated within the liver. Secondarily, as opposed to the simple setting of PH of instant liver loss and hypertrophy as the exclusive adaptive response, the twin processes of PVO are closely co-regulated, since the development of hypertrophy is strictly bound to concomitant atrophy^6^. Therefore, PVO techniques exploit the different adaptive mechanisms of portally occluded- and non-occluded liver lobes. The latter undergo a peculiar regenerative process, presenting a volume increase, presumably accounting for the lost capacities of lobes devoid of portal blood. However, beyond well-documented morphological alterations, there is scarce evidence on how PVO procedures affect cellular and organ-wide hepatic functions, despite their paramount basic research and clinical significance^10^. Although a ‘full-scale’ hepatic functional quantification remains a platonic idea, the analysis of organic anion (bilirubin, bile salts, etc.) transport may be fairly representative, judging from its central physiological and clinical relevance^11^. On one hand, this may be well performed in *in vitro* cell cultures, which provide basic information regarding the specific characteristics of the involved transport routes. However, culturing intrinsically discards dead cells, limiting data interpretation in living systems. Therefore, *in vivo* measurements are essential for the evaluation of alterations in liver functions. Global tests, such as the clinically widely applied indocyanine-green (ICG) clearance test, only measure total liver function and are insufficient during PVO procedures, when liver function is inhomogeneously distributed between the atrophic and hypertrophic lobes. However, recent state-of-the-art nuclear imaging technologies, such as hepatobiliary scintigraphy (HBS) and single photon emission tomography (SPECT) using liver-specific radiopharmaceuticals (like ^99m^Tc-mebrofenin [^99m^Tc-2,4,6-trimethyl-3-bromo iminodiacetic acid]) enable sophisticated functional assessment of individual liver regions including the critically important FLR^12,13^. Furthermore, liver function may also be visualised by the novel fluorescence-based method, ‘confocal laser endomicroscopy’ (CLE)^14^. Conclusively, our goal was the temporal assessment of morpho-functional aspects of PVO-induced LR with a special focus on organic anion transport, involving *in vitro*, as well as multimodal *in vivo* analyses. Additionally, we wished to clarify the role that cellular junction proteins and organic anion transporters play in the regenerative potential of ligated lobes undergoing involution.

## Results

### PVL induces both atrophy and hypertrophy with unchanged total liver weight

**Figure 1:**
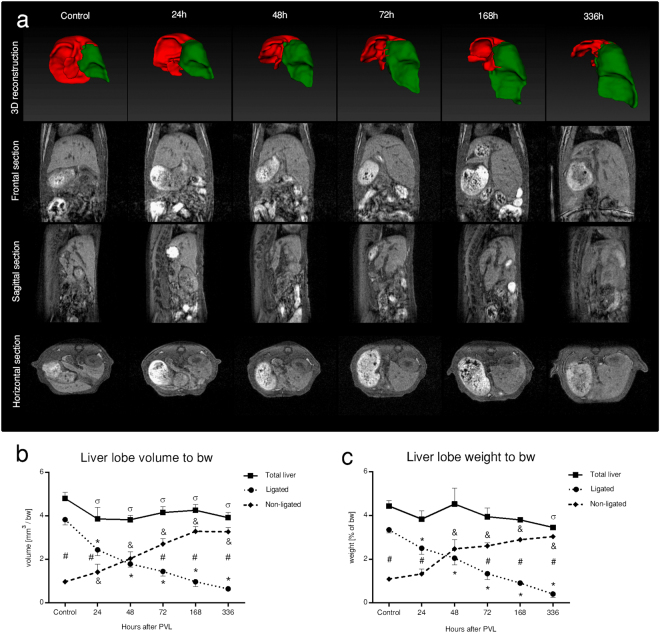
Macroscopic changes. Portal vein ligation (PVL) mediated the progressive atrophy and hypertrophy of ligated- and non-ligated lobes, respectively, which was reflected on all (frontal, sagittal, and horizontal) sections as well as three-dimensional (3D) reconstructions of serial magnetic resonance imaging scans (**a**). Quantification with liver lobe volumetry (bw: body weight) and weighing of liver lobes collected in parallel also confirmed the atrophy-hypertrophy complex, whereas essentially unchanged parameters of the total liver marked the balanced nature of the process (**b**,**c**). Statistical analysis was performed with analysis of variance (ANOVA) with Bonferroni’s *post hoc* test to correct for multiple comparisons. Results are given as means ± standard deviation, with (**a**,**b**) representing n = 9 and ***(C)*** representing n = 5 animals per time points. ^‘*’, ‘&’^ and ^‘σ’^ indicate p < at least 0.05 for actual versus control values of ligated lobes, non-ligated lobes or total liver, respectively. ^‘#’^ indicates p < at least 0.05 ligated lobes versus contemporary non-ligated lobes.

As the cornerstone for further analysis, macroscopic parameters of regeneration were initially determined (Fig. 1). The weight of the ligated lobes (LL) significantly decreased (0.41 ± 0.17% vs. 3.41 ± 0.14%), whereas non-ligated lobe (NLL) weights significantly increased (3.04 ± 0.1% vs. 1.09 ± 0.1%) as compared to control. Total liver lobe weight remained essentially unchanged. *In vivo* liver lobe volumes following PVL were also assessed in the mebrofenin group by serial postoperative MRI scans. Accordingly, 3D-reconstructions of liver lobe volumes showed close correlation with the weight of both the LL (r = 0.976; p = 0.001) and NLL (r = 0.96; p = 0.002). In general, PVL has proven viable in mediating the established atrophy-hypertrophy complex of the liver.

### Hepatocytes from both lobes unequivocally maintain polarity and bile duct formation capacity, while gradually recovering from a temporary functional decrease

**Figure 2:**
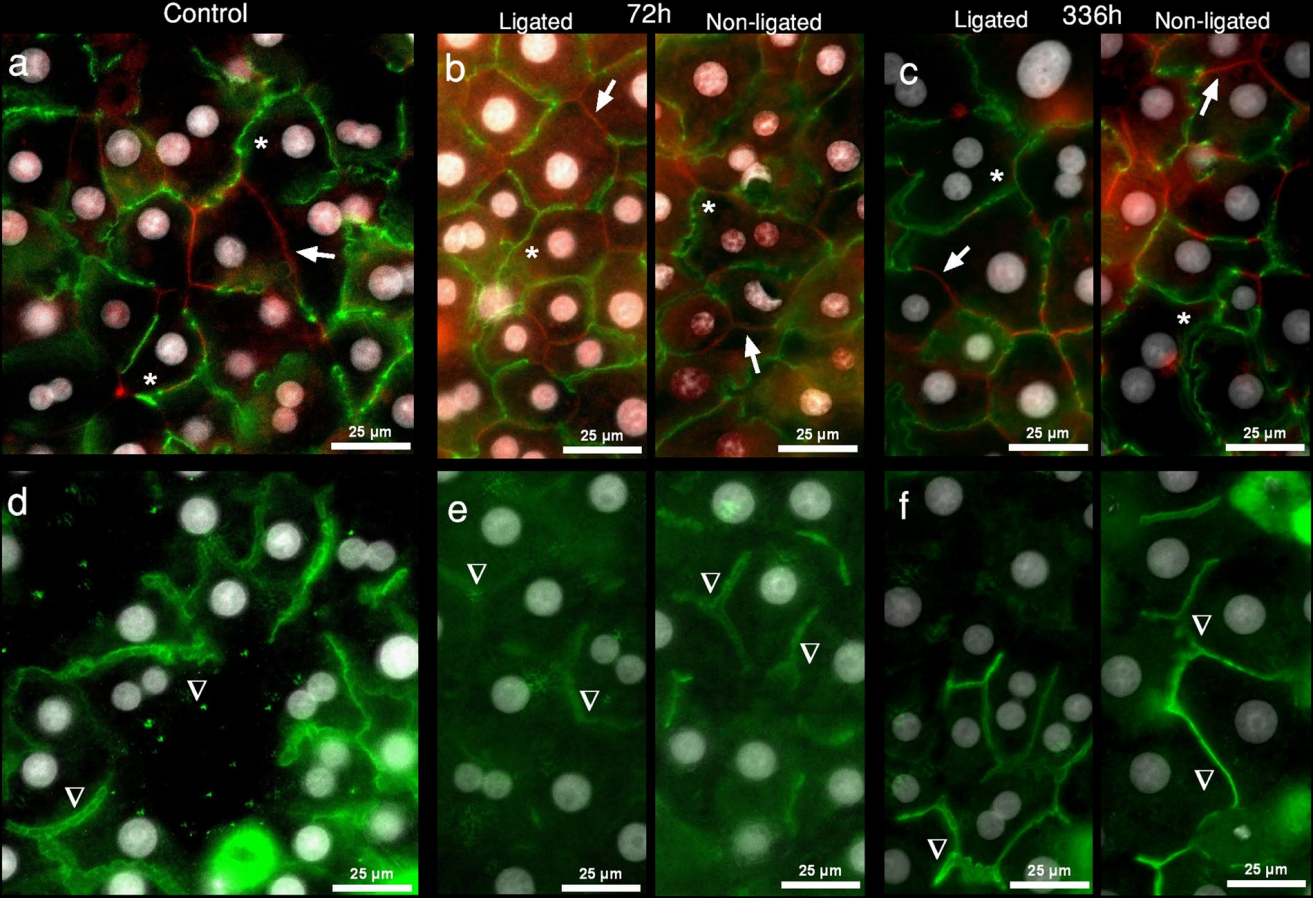
*In vitro* immunofluorescence. Representative images of cultured hepatocytes isolated from liver of control rats, as well as from the ligated and non-ligated lobes of rats 72 h and 336 h after portal vein ligation are shown. The samples were immunostained for the sodium-taurocholate cotransporting polypeptide (Ntcp) [labelled as red] and zonula occludens-1 (ZO-1) protein [labelled as green] (**a**–**c**), as well as for the bile salt export pump (Bsep) [labelled as green] (**d**–**f**). Cells, regardless of lobar or temporal origin, maintained their ability of generating viable cultures, forming bile canaliculi correctly sealed with ZO-1, and properly expressing the essential hepatic transporters, Bsep and Ntcp. Cell nuclei stained with DAPI (4′,6-diamidino-2-phenylindole) are show in grey scale. Representative markings indicate ZO-1 positive intercellular connections (asterisks), membrane expression of Ntcp (arrowheads) (**a**–**c**), as well as specific bile canaliculi (triangles) (**d**–**f**). White bars represent 25 µm. The whole sets of pictures from all time points are available online in Supplementary Information (Supplementary Figures 1, 2).

**Figure 3:**
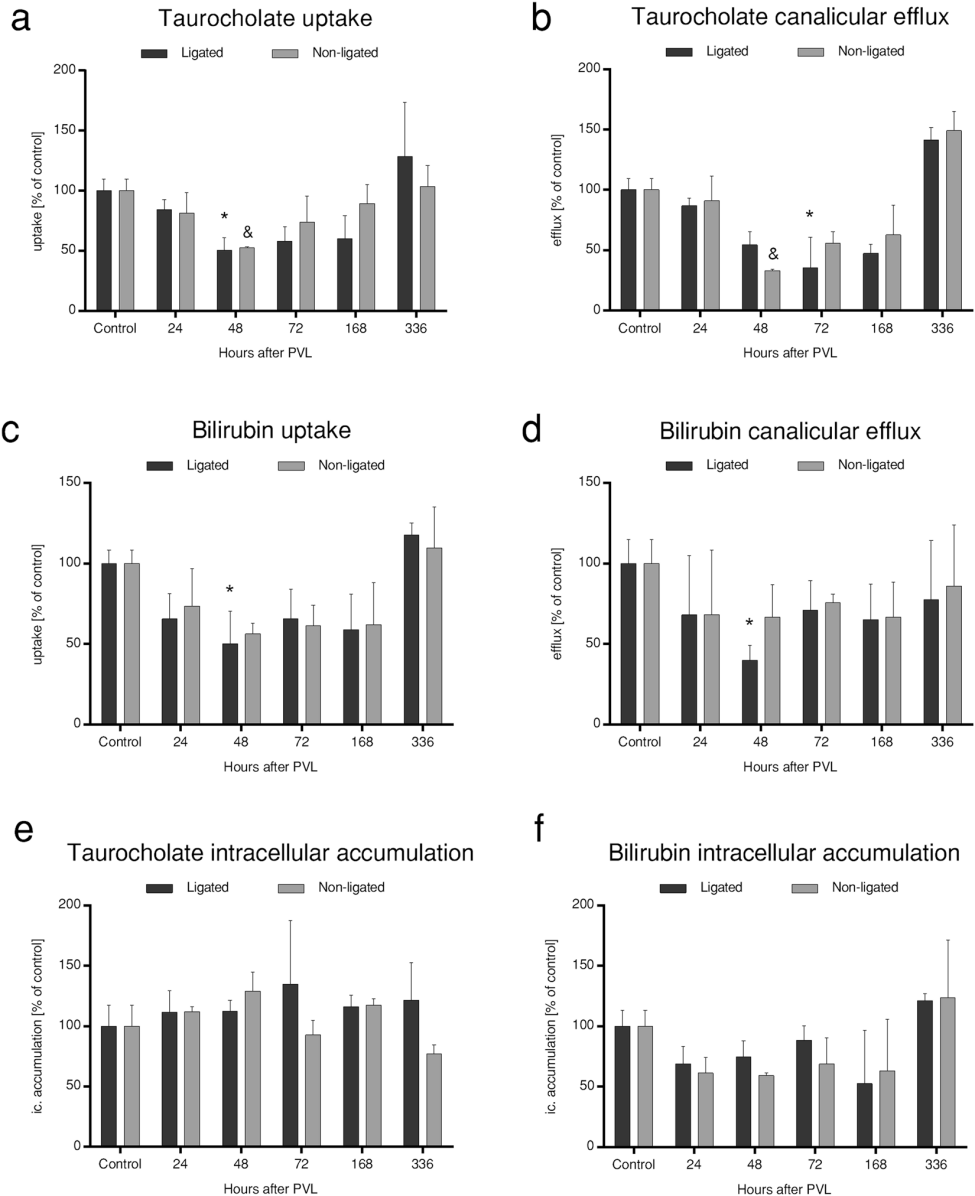
*In vitro* transport analysis. The specific taurocholate (**a**,**b**,**e**) and bilirubin (**c**,**d**,**f**) transport of hepatocytes 72 h in cultures isolated from ligated- and non-ligated liver lobes were measured *in vitro*. The uptake and excretion of both substances were uniformly reduced at 48–72 h in both lobes, normalising thereafter by 168–336 h. While changes in terms of ligated lobes were significant, the bilirubin transport of non-ligated lobes was only tendentiously decreased, implying a more preserved state (**a**–**d**). As a result of adaptive patterns, the intracellular compartment of both compounds remained unchanged (**e**,**f**). PVL: portal vein ligation. Statistical analysis was performed with analysis of variance (ANOVA) with Bonferroni’s *post hoc* test to correct for multiple comparisons. Results are given as means ± standard deviation, with n = 5 animals per time points. ^‘*’^ and ^‘&’^ indicate significant differences in the transport rates of ligated lobes and non-ligated lobes, respectively, as compared to control values (p < 0.05).

For the basic evaluation of morphological and functional attributes of cells constituting the LL and NLL, corresponding cell cultures were created. A substantial number of viable hepatocytes (at least 50 million) could be isolated at all time points from both the NLL and LL. Immunofluorescence (IF) staining for zonula occludens-1 (ZO-1), sodium-taurocholate cotransporting polypeptide (Ntcp), and bile salt export pump (Bsep) demonstrates that isolated cells from both liver regions are able to generate polarised cultures with normal morphology, forming bile canaliculi, expressing and properly localising the key liver transporters (see Fig. 2 for representative images, IF for all time point are shown in Supplementary Figures 1, 2). To assess *in vitro* function beyond morphology, taurocholate and bilirubin transport measurements were performed (Fig. 3). Taurocholate (TC) uptake and canalicular efflux were significantly deteriorated at 48–72 h in both lobes. Likewise, the uptake and canalicular excretion of bilirubin were also significantly reduced at 48 h in LL. Considering the bilirubin transport of NLL, a very similar, though non-significant tendency was detected. However, intracellular load of both TC and bilirubin remained constant throughout the observation period. Results of the *in vitro* analysis confirmed the constant presence of viable and transporter-expressing cells in both the LL and NLL, as well as a transient, but marked reduction of TC and to a lesser extent bilirubin transport in both lobes without significant intracellular accumulation.

### Temporary bilobar reduction of transporter expression with necroapoptotic patches in ligated lobes as well as divergent interlobar acinar changes

**Figure 4:**
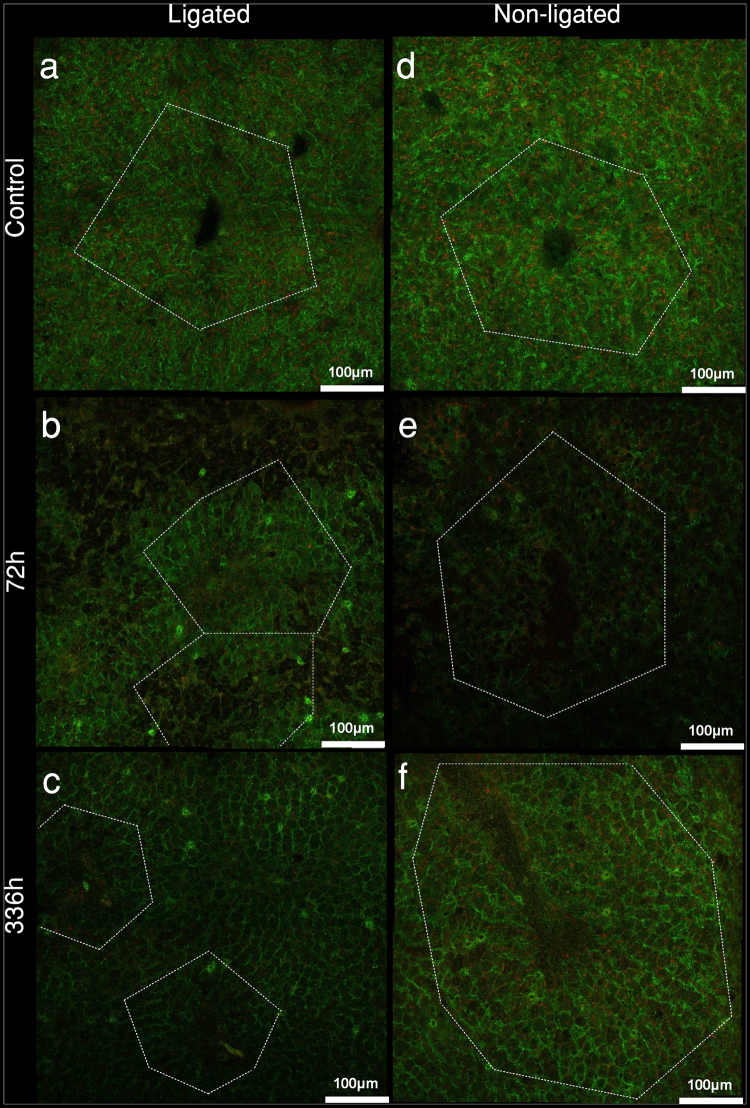
Morphology of *ex vivo* liver sections. To characterise acinar structure and transporter expression after portal vein ligation, immunofluorescence staining for sodium-taurocholate cotransporting polypeptide (Ntcp) [labelled as green] and canalicular multispecific organic transporter (Mrp2) [labelled as red] were performed on sections of snap frozen liver specimens excised from ligated lobes (**a**–**c**) and non-ligated lobes (**d**–**f**). As compared to control (**a**,**d**), 72 h sections of both lobes showed lower transporter expression with void patches in ligated lobes (**b**). Transporter expressions at 336 h were bilaterally re-established; however, diverging acinar changes were observed as shrunk and enlarged liver acini were seen in ligated (**c**) and non-ligated lobes (**f**), respectively. Dashed line markings represent arbitrary acinar borders. White bars represent 100 µm.

IF staining carried out on frozen sections of the LL and NLL of control rats demonstrated normal acinar outlines with proper canalicular and basolateral localisation of canalicular multispecific organic transporter (Mrp2) and Ntcp, respectively (Fig. 4). 72 h after PVL, the LL displayed large patches with abrogated staining for Mrp2 or Ntcp, likely corresponding to necroapoptotic lesions. Meanwhile NLL sections at 72 h lacked any regional differences; however, expression level of both proteins was generally reduced. After 336 h, transporter expression was restored in both lobes. However, acinar structures became divergent; LL acini were markedly shrunk, while NLL acini became expanded (representative hematoxylin-eosin stained sections are shown on Supplementary Figure 3).

### Lobe performance rearrangement maintains bilirubin excretion and keeps serum bilirubine in control

**Figure 5:**
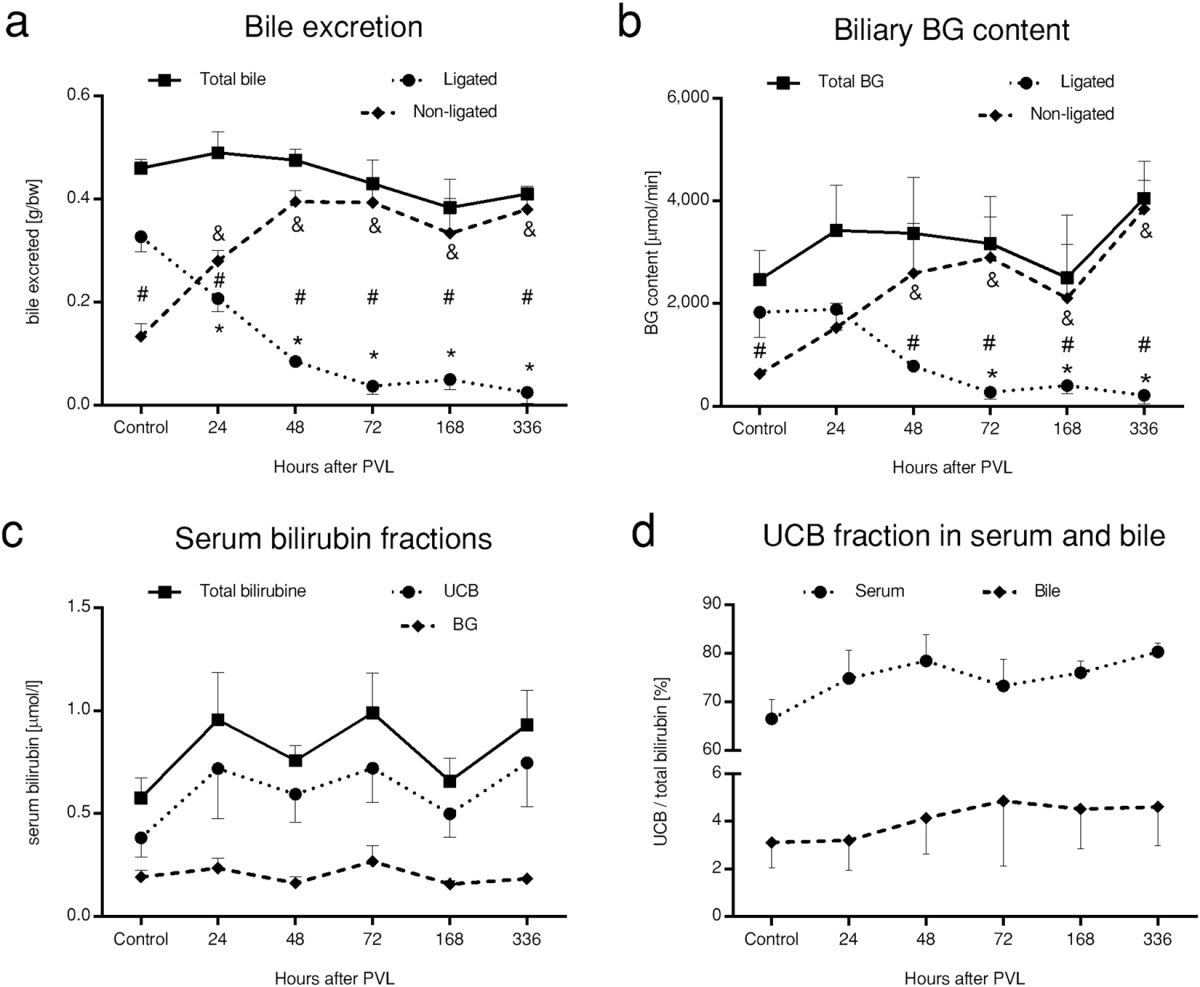
Bile excretion and bilirubin homeostasis. Acquired via selective biliary drainage, bile (**a**) and bilirubin glucuronide (BG) (**b**) excretion mirrored the atrophy-hypertrophy complex induced by portal vein ligation (PVL), with progressively decreasing output of ligated lobes and the definite gain-of-function of non-ligated lobes, while total excretion remained unchanged. Serum bilirubin levels (**c**), as well as the serum and biliary fraction of unconjugated bilirubin (UCB) to total bilirubin (**d**) were unaffected. Statistical analysis was performed with analysis of variance (ANOVA) with Bonferroni’s *post hoc* test to correct for multiple comparisons. Results are given as means ± standard deviation, with n = 5 animals per time points. ^‘*’^ and ^‘&’^ indicate significant differences in the values of ligated lobes and non-ligated lobes, respectively, as compared to control values (p < 0.05).

To assess *in vivo* bile excretion, selective biliary drainage of the LL and NLL was performed (Fig. 5). The bile output was progressively decreased, and oppositely, significantly increased in the LL and NLL, respectively, with unchanged overall bile output. Total biliary BG contents altered in parallel with bile production. BG output showed a bilateral response in accordance with atrophy-hypertrophy. There were no significant changes in serum levels of either unconjugated bilirubin (UCB), conjugated bilirubin (BG), or total bilirubin in any time points, despite their tendentious rise. In line with this, both the serum and biliary UCB to total bilirubin ratios were unchanged. Altogether, both bile and BG excretion indicated bilateral responses in basic hepatic function, involving the functional gain of NLL and the continuous decay in LL function. Meanwhile serum- and biliary levels of bilirubin fractions were unchanged, implying that elimination of endogenous substrates is unaffected following PVL.

### Transient suppression of global hepatic function with the lag of excretion

**Figure 6:**
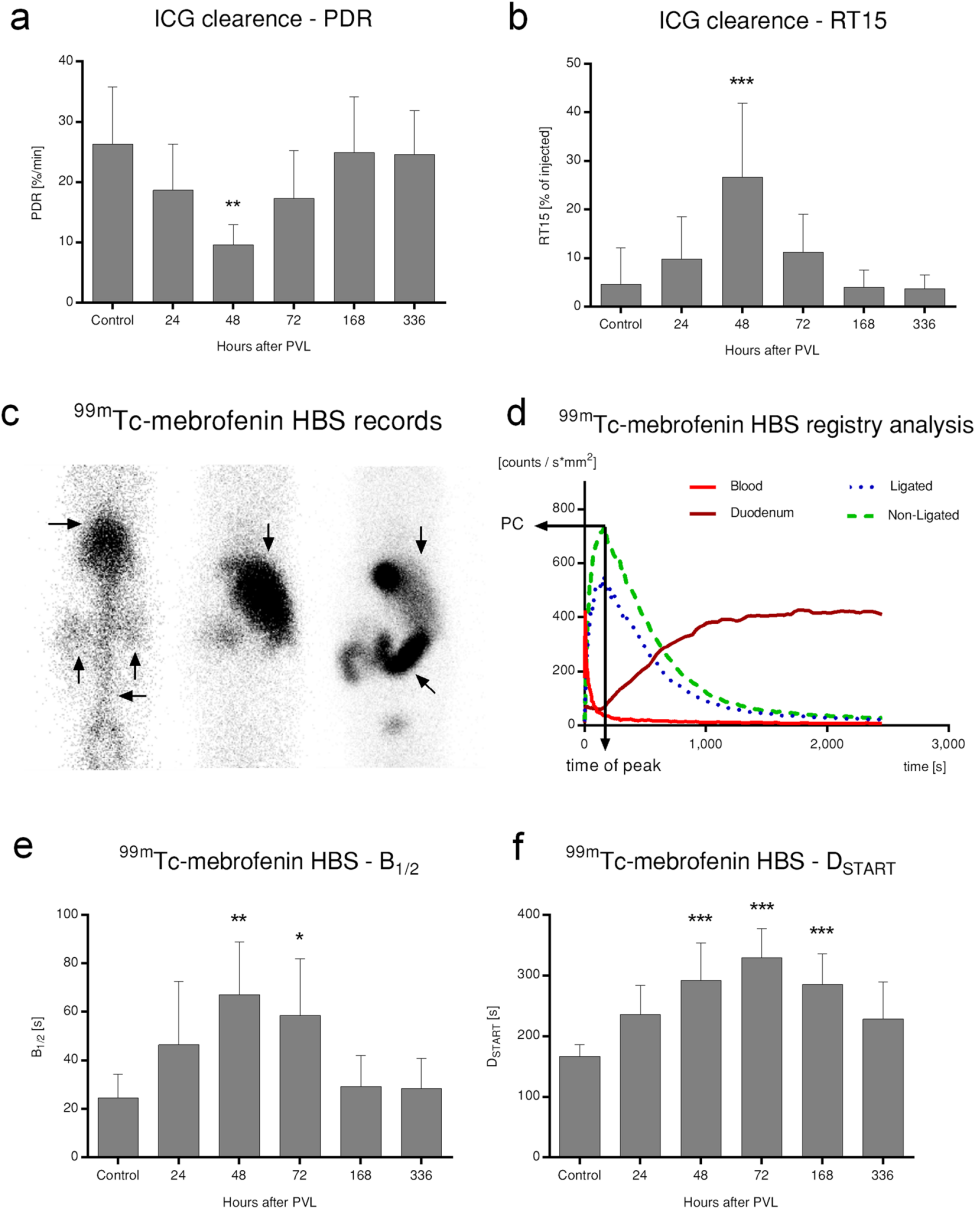
Global liver function. Total *in vivo* hepatic function after portal vein ligation (PVL) was evaluated by the indocyanine-green (ICG) clearance test (**a**,**b**) and planar ^99m^Tc-mebrofenin hepatobiliary scintigraphy (HBS) (**c**–**f**). A significant temporary reduction in global function at 48 h was reflected in decreased plasma disappearance rate (PDR) of ICG ***(A)***, leading to increased ICG retention in the bloodstream at 15 minutes (RT15) (**b**). Planar ^99m^Tc-mebrofenin HBS was recorded in three dynamic phases illustrated on section ***c***, where the first *(left-to-right)* snapshot corresponds to tracer injection and rapid distribution, the second confirms localisation to the liver, whereas the third exhibits hepatic excretion and the intestinal phase. Different organs are marked with pointing arrowheads as follows: rightward - heart, upward - kidneys, leftward - abdominal aorta, downward - liver, and oblique - intestine(s) (**c**). Evaluation was performed by region-of-interest allocation to heart, duodenum, as well as ligated and non-ligated lobes, producing intensity curves with different characteristics on HBS registries, including exponential decay for the bloodstream, and a sigmoid saturation-like curve for the duodenum. Ligated and non-ligated lobe functions were judged by their peak counts (PC), as well as derived ratios (**d**). Similar to the ICG clearance test, global hepatic uptake (B_1/2_) and excretion (D_START_) parameters also indicated a transitional reduction in global liver function at 48–72 h. In contrast to global hepatic uptake, the normalisation of global hepatic excretion lagged until 336 h (**e**,**f**). Statistical analysis was performed with analysis of variance (ANOVA) with Bonferroni’s *post hoc* test to correct for multiple comparisons. Results are given as means ± standard deviation, with (**a**,**b**) representing n = 7 and (**c**–**f**) representing n = 9 animals per time points. ^‘*’/‘**’/‘***’^ indicate significant differences as compared to control values (p < 0.05/0.01/0.001, respectively).

To assess *in vivo* global hepatic functions dynamic liver function tests were performed (Fig. 6). The ICG-clearance test exhibited a marked drop between 24–72 h (diminished plasma disappearance rate [PDR], 5.8-fold increased retention at 15 minutes [RT15]), which rapidly normalised by 168 h. These results were echoed by the hepatobiliary scintigraphy (HBS) test. Blood half-life (B_1/2_), reflecting global hepatic uptake function, exhibited a very similar pattern to RT15, as significantly increased at 48–72 h, and rapidly normalised thereafter. First duodenal appearance (D_START_)_,_ reporting on global hepatic excretory function showed similar changes; noteworthy though, that it was significantly prolonged at 168 h, and was not completely restored by 336 h. Taken together, contrary to the excretion of endogenous organic anions such as bilirubin, the elimination of exogenous ligands suffered a temporary reduction, indicating a transient suppression in global liver function.

### Compensatory increase of xenobiotic excretion in the non-ligated lobes complements permanently deteriorated ligated lobe functions

**Figure 7:**
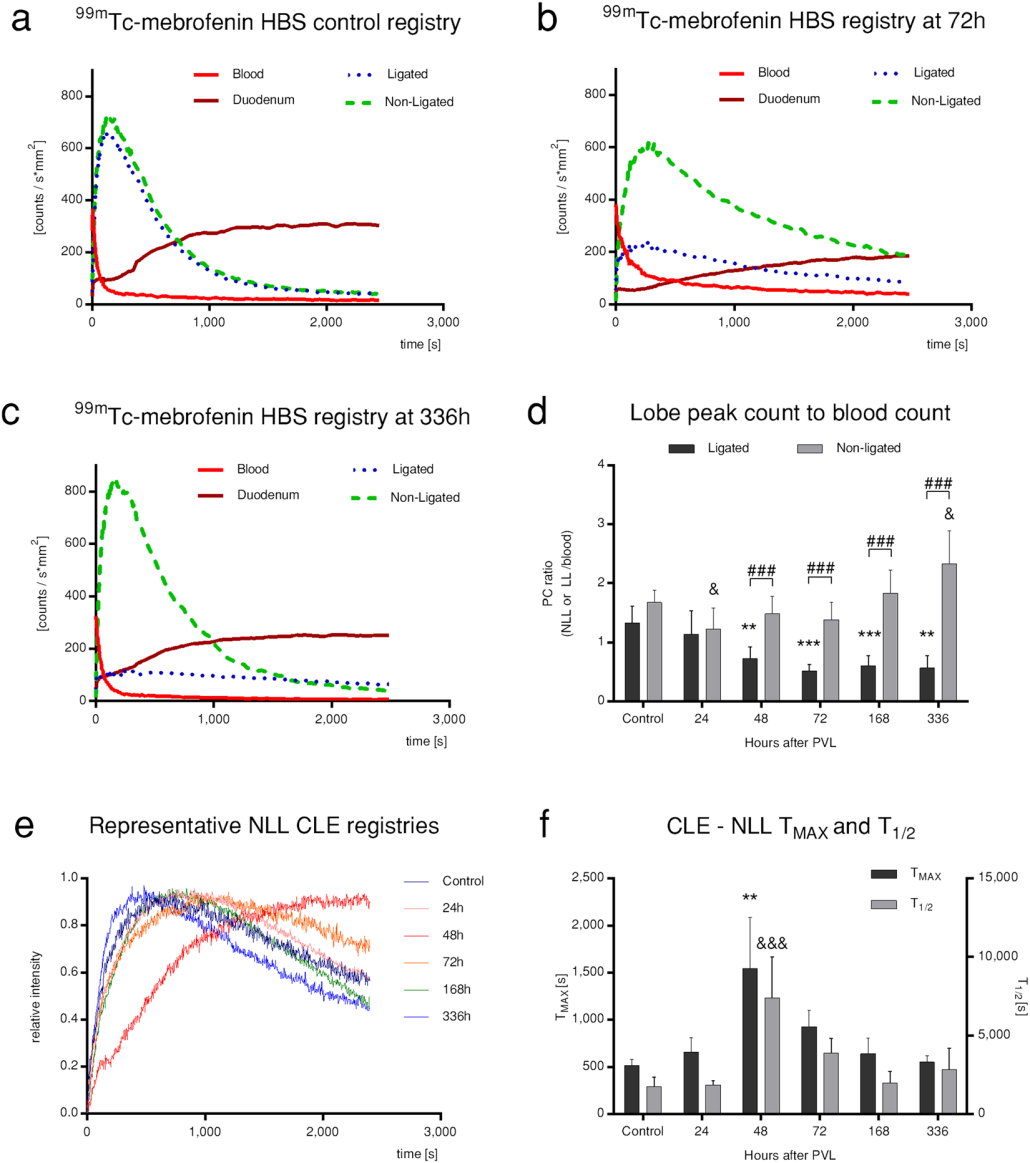
Regional liver function. Planar ^99m^Tc-mebrofenin hepatobiliary scintigraphy (HBS) (**a**–**d**) and confocal laser endomicroscopy (CLE) (**e**,**f**) were utilised to assess regional hepatic function of both ligated lobes (LL) and non-ligated lobes (NLL) after portal vein ligation (PVL). As compared to control conditions (**a**), ^99m^Tc-mebrofenin HBS registries at 72 h showed diminished regional function reflected in peak reduction and widening (**b**). However, according to 336 h registries, these changes were reversed in the NLL, but further deteriorated in the LL (**c**). Hence, the divergence of lobar functional output was confirmed by a progressively developing gap between NLL and LL curves, attributable to a solid functional gain of the NLL, as reflected by the ratio of lobar peak counts (PC) per corresponding blood counts (**d**). CLE intensity curves of indocyanine-green transport revealed altered curves at different time points of liver regeneration (**e**). Following curve analysis, the transitional elevation and subsequent normalisation of derived parameters (T_MAX_, T_1/2_) proved a transient suppression and rapid regeneration of NLL uptake and excretory functions (**f**). Statistical analysis was performed with analysis of variance (ANOVA) with Bonferroni’s *post hoc* test to correct for multiple comparisons. Results are given as means ± standard deviation, with (**a**–**d**) representing n = 9 and (**e**,**f**) representing n = 5 animals per time points. ^‘*’/‘**’/‘***’^and ^‘&’/‘&&’/‘&&&’ ^indicate significant differences in the ligated lobes or non-ligated lobes, respectively, as compared to control values, whereas ^‘#’/‘##’/‘###’^ indicate significant differences between the ligated and non-ligated lobes (p < 0.05/0.01/0.001).

Nuclear imaging techniques enable sophisticated evaluation of regional liver function. Therefore, a planar ^99m^Tc-mebrofenin HBS test was performed to assess hepatic function separately in the LL and NLL (Fig. 7). As illustrated by representative HBS registries, the peaks in both LL and NLL decreased and widened after PVL. However, by 336 h, the kinetics of drug elimination normalised in the NLL, whereas flattened in LL resulting in a solid gap between their curves. Accordingly, regional function was well-reflected in relative ratios of the LL and NLL peak counts (PC) to the corresponding blood count. A transitional regional functional depression was observed in both lobes at 24–72 h, which was gradually restored in the NLL at 336 h, but remained diminished in LL, resulting in a significant difference between LL and NLL regional functions after 48 h. To further elaborate this, we selectively measured NLL regional function by CLE, which allowed the monitoring of ICG transport in a particular liver region. Time of maximum (T_MAX_) (~uptake) and half-life (T_1/2_) (~excretion) values, derived from intensity curves, confirmed a transitional drop of NLL regional uptake and excretory function at 24–72 h, after which both parameters gradually reached preoperative levels. Altogether, our observations indicate a transient, bilateral deterioration of regional function, followed by the compensatory functional gain of the NLL resulting in a massive divergence of liver function.

## Discussion

Portal vein ligation-induced liver regeneration remains an essential element of arsenal in liver surgery for the curative two-stage resection of primarily unresectable liver tumours. Therein, hepatic functional aspects of this procedure are of great significance. Hence, the present experimental analyses dedicated prime interest to the temporal alterations of both global and regional liver function by ‘benchmarking’ cellular aspects with culturing *in vitro* and assessing hepatic function with relevant modern imaging technologies *in vivo*. Remarkably, despite the absence of portal inflow and its special blood-borne constituents (nutrients, hormones, and growth factors, etc.), and the severe morphological consequences of atrophy, such as necroapoptosis and structural remodeling, we found evidence for the presence of viable, transporter-expressing and functioning hepatocytes in the LL even as far as 336 h after PVL, implying the plausibility of process reversibility. We have further confirmed a transient drop in global hepatic function, followed by a relatively rapid recovery. In the background of re-establishment, a clear divergence of *in vivo* regional hepatic function was found, with progressive involution of the LL and the massive functional gain of the NLL, leading to inhomogeneous functional distribution between liver lobes and a shift in regional function towards the NLL. Finally, the present study is the first known application of confocal laser endomicroscopy for the analysis of segmental hepatic function, confirming the temporary deterioration of NLL uptake and excretory functions.

Perplexingly, the origination of all these widespread changes may eventually be traced to PVL. The mere ligation of the portal vein branch is ultimately responsible for overwhelming alterations of both hepatic structure and physiology. Hemodynamically, liver lobes become separate after PVL, with a halved total-, and an increased fraction of arterial blood flow (hepatic artery buffer response - HABR: increase in arterial flow due to the decrease in portal flow leading to a decreased ‘washout’ of vasodilatative adenosine) through the LL, meanwhile, a hyperperfusion (~230%) of mainly (>97%) portal blood flow with reverse HABR takes place in the NLL^6^. Nevertheless, splanchnic circulatory redistribution is inherently accompanied by a reduced oxygen supply of both hepatic lobes, as well as an increased delivery of portal constituents to the NLL such as nutritional factors, recirculating bile acids, hormones, and growth factors, while these portal blood-borne factors are dramatically less accessible for the LL^6^. As a result of these complex hemodynamic, hypoxic, and humoral changes, liver regeneration is triggered in the NLL, while atrophy with necroapoptotic cell death, acinar atrophy, and pseudocapillarisation takes place in the LL.

In the present study, the initial assessment of macroscopic parameters served as a proof of concept and model reliability. To no contention of our previous results and literature data, liver lobe weight and volume both confirmed the development of LL atrophy and NLL hypertrophy. Beyond this, we have earlier found extensive necroapoptotic lesions in the LL and an intensive rise in NLL mitotic activity^15,16^. Total liver weight remained unchanged, indicating the balanced nature of the process. Having verified model reproducibility, next we embarked to explore functional aspects of PVL-induced LR. With respect to the inexplicable complexity of ‘liver function’ an all-around functional assessment remains an idealistic, yet impossible approach. Therefore, we evaluated liver organic anion transport (involving Ntcp, Oatp, and Abc transporters), for it fulfils a critical role in hepatic detoxification pertaining to both endogenous (bilirubin metabolites and bile acids) and a wide range of exogenous substrates (drugs and other xenobiotics)^11^. The latter further underlines its key importance, for on one hand, transporter activity disturbances massively alter clinically administered drug pharmacokinetics. On the other hand, several clinically used liver function tests (ICG-clearance, HBS, functional MRI) are methodically and pharmacokinetically based on specific organic anion transport routes^11,17^. The current study initially focused on the elementary, cellular aspects of PVL-induced functional changes *in vitro* by cell culturing. Hepatocytes with overall cell viability of >90%, with a sustained potential for generating viable cultures with adequate cell junction and bile canaliculus formation, as well as proper expression and localisation of Ntcp and Bsep transporters could be isolated from both lobes at any time points. Furthermore, the quantitative transport function of cell cultures from either liver segments were measured *in vitro*. The canalicular efflux of taurocholate, mediated by Bsep in an ATP-dependent manner, was transitionally depressed at 48–72 h in both lobes, which may be explained by cellular energetic imbalance. While lowered energy status in the LL might be due to decreased oxygen delivery secondary to the drastically reduced total blood flow, in the NLL, it is presumably a consequence of the intense proliferation, peaking around 48 h, which idea is supported by our previous findings^15^, as well as other reports, stating a decreased cellular energy charge in the initial phase of LR following PVL^18,19^. TC uptake showed similar transitional depression. Besides energetic issues, an adaptive response preventing intracellular bile acid overload toxicity is also suspected, since following PVL, all intestinally reabsorbed, portal-borne TC is directed towards the NLL^20^. Accordingly, TC uptake decreased while basolateral efflux tendentiously increased (see Supplementary Figure 5), resulting in unchanged intracellular TC levels. A similar adaptive response was previously reported during PH-induced LR^21^. Pattern of changes in bilirubin transport was similar; however, alterations remained non-significant, implying a more tightly regulated hepatic bilirubin transport. A possible explanation might lie in the higher variability, redundancy and lower transporter-dependency of bilirubin uptake transport as compared to TC transport. Oatp1a/1b knock-out mice models revealed that besides Oatp1a1, -1a4, and -1b2, bilirubin and UCB may also enter hepatocytes by bromosulfophthalein/bilirubin-binding protein-mediated transport and passive basolateral diffusion, respectively. Moreover, loss-of-function of either transporter may be well compensated. Meanwhile, bile acids are predominantly imported by Oatp1a, -1b2, and Ntcp, and functional deterioration cannot be fully compensated by the other transporters^17,22^. Altogether, *in vitro* results imply a preserved functional status, which seems well-established in terms of the NLL. However, it is of paramount interest, that even after 336 h, the LL still contains viable and functional cells. Regarding this, the question arises, whether these cells were capable of re-developing hepatic acinar structure and specific function, if allowed portal reperfusion with the cessation of occlusion. Until recently, adaptive changes after PVO were consensually regarded as irreversible with clear endpoints of atrophy and hypertrophy. However, the utilisation of absorbable embolization materials led to the discovery of reversible PVO. The reversibility of occlusion and subsequent macroscopic changes have been reported^23,24^. Our findings significantly add to the cellular background by testifying that in idealised circumstances (cultures), hepatocytes residing in the atrophic LL are not only capable of forming viable cultures, but also express hepatocyte-specific transporters, whose functionality is demonstrated by *in vitro* organic anion transport. These open new insights of LR by confirming the maintained viability of hepatocytes in pathophysiological conditions, capable of stimulation for re-establishment of liver structure and function. The latter may have key significance regarding both basic research and clinical fields of regenerative and transplantation medicine.

Since the applicability of cell culturing techniques are limited by the fact that viable cells are selectively isolated and investigated, these *in vitro* approaches cannot assess the degree and functional consequences of cell death, which is unambiguously present following PVL. Therefore, *in vitro* experiments were complemented with *ex vivo* and *in vivo* methods to assess conditions in living organisms. To investigate morphology and zonal transporter expression, Ntcp and Mrp2 IF were performed on snap-frozen liver sections. Accordingly, 72 h sections of the LL revealed void patches, short of transporters, likely reflecting necroapoptotic regions, absent from contemporary NLL sections, displaying generally lower expression of both transporters. Meanwhile at 336 h, LL and NLL liver acini were shrunk and enlarged, respectively, with no void patches. Alterations in LL and NLL liver acini morphology are supported by representative hematoxylin-eosin stains (see Supplementary Figure 3) and our earlier liver resin perfusion findings^15^. Overall, a temporarily lower transporter expression was confirmed in both lobes, which may contribute to the contemporary suppression of transport functions.

Afterwards, we focused on *in vivo* function. The proximal element was the evaluation of bile- and bilirubin excretion through selective biliary drainage for it well reflects basic aspects of hepatic function due to its high energy consumption. Both bile- and conjugated bilirubin output mirrored changes in liver lobe weights, inasmuch as bile- and BG production of the LL was progressively deteriorated, while a massive NLL output gain was observed, with preserved overall bile- and BG excretion. Meanwhile, serum levels of UCB, BG, and total bilirubin; as well as the fraction of UCB to total bilirubin in serum and bile were unchanged, corroborating with *in vitro* results, indicating a maintained bilirubin transport function of the liver. The preserved excretion of the endogenous, yet toxic agent bilirubin might be a highly important defensive mechanism. All the same, upon its low sensitivity for fine changes in hepatic function, clinical reports pronounced serum bilirubin levels – even as part of clinical scoring systems - inaccurate for liver functional prediction^12,15^. Therefore, dynamic tests were performed, starting with the clinically most frequently applied liver functional test to date. The ICG-clearance test assesses overall liver function, based on the blood disappearance of the fluorescent dye ICG, which is exclusively eliminated by the liver without metabolism and enterohepatic recirculation, via a similar transport pathway to several exo- and endogenous agents^12^. Our results indicated a transient reduction in global liver function between 24–72 h despite the lack of changes in serum bilirubin levels. Unfortunately, the ICG-clearance test is only informative on whole liver function, greatly limiting its applicability. In contrast, the nuclear imaging method ^99m^Tc-mebrofenin HBS enables the anatomical localisation of functional data through radioactive tracer signal detection from any parts of the body, allowing the differentiation of hepatic uptake and excretion, as well the selective measurement of segmental liver function^12,13^. Clinically, it was found to accurately measure FLR function (close correlation between preoperative- and postoperative scans)^25^, predict posthepatectomy liver failure^26^, as well as allow the functional itineration of the second-stage liver resection^27–29^. For these reasons, ^99m^Tc-mebrofenin HBS is a highly relevant method for the quantitative assessment of (regional) liver function, currently considered as one of the most accurate and sophisticated over other, previously known tests^13,28,30^. Reports on either experimental or clinical liver functional analysis are available in the settings of PH^30^, PVE^10^, and the new method associating liver partition with portal vein ligation for staged hepatectomy (ALPPS)^27–29^. However, to our knowledge, no complex or comparative analysis is available on the specific characteristics of liver function utilising the above-mentioned imaging methods regarding LR induced by PVL, even though the latter is still widely applied worldwide. Our results concerning global hepatic uptake and excretion echoed the ICG-clearance test results, confirming a temporary depression of global liver functions. Interestingly, global uptake normalisation was complete by 168 h, while global excretion recovered only by 336 h. A hypothetical explanation for this lag could be the higher energy consumption of (primarily active) canalicular excretion versus (facilitated) basolateral uptake transport parallel to a lower intracellular energy charge. However, its contribution at 168 h is presumably limited when cells are regaining normal energetic levels following the cessation cell proliferation^18,19^. Instead, mechanical factors may play a more significant part, for PVL leads to intraabdominal adhesions as well as markedly different anatomical-postural changes of liver lobes, as seen on MRI images, which may also interfere with tracer duodenal appearance. In addition, the planar ^99m^Tc-mebrofenin HBS performed in the present study also provided quantified information on the individual functional aspects of the LL and the NLL. Following an initial reduction in both lobes, we found a progressive divergence of regional hepatic function, with the permanent depression of LL and a massive gain in NLL function, resulting in a functional shift towards the NLL. In the wake of experienced dramatic changes in NLL function, we embarked to further investigate its performance with confocal laser endomicroscopy. CLE is a novel optical imaging method with an endoscopic wire probe, primarily designed for non-invasive ‘live optical biopsy’ and improved decision making during clinical endoscopy for gastrointestinal and bronchoalveolar diseases^14^. From the wide variety of CLE application types, a few reports are available regarding the liver^31,32^. However, to the best of our knowledge, our study is the first known experimental application of CLE in the setting of selective probe-measurement of NLL regional function. Therein, a transient depression in both uptake- and excretion intensities was observed between 24–72 h, which normalised by 168 h, corroborating the results of the ^99m^Tc-mebrofenin HBS concerning NLL function. Regarding future concepts, percutaneous realisation of regional liver functional probing by CLE in patient care might have great clinical significance, for it could improve or extend the available diagnostics for the chronic or preoperative analysis of liver function.

The combined results of all *in vivo* functional tests imply that recovery of global liver functions is attributable to the massive functional gain of the NLL, while LL function is permanently deteriorated. Thus, there is an apparent discrepancy with *in vitro* results demonstrating similar functional capacities of cells from the LL and NLL. This is most likely stemmed from the fact that *in vitro* approaches apply standardised conditions of culture, nutrition and substrate provision, with no effluent, structural or physiological interfering factors. Additionally, selective isolation of viable cells for *in vitro* methods hinders studying cell death-related alterations. Meanwhile, during *in vivo* experiments, function is measured under (patho)physiological conditions, with dramatic hemodynamic and histological changes. Normally, hepatic endothelial cells are abundantly fenestrated to allow an excessive exchange of substances between sinusoids and the space of Disse^33^. However, PVL leads to the arterialisation of the LL, with respect not only to blood inflow, but also by the partial transition, or ‘pseudocapillarisation’ of hepatic sinusoids into standard capillaries, with reduced number of endothelial fenestrations, increased perisinusoidal fibrosis, and the development of a basal lamina-like fibroid layer^33^. Furthermore, according to our earlier results^15^, and current *in vivo* IF, shrinkage of LL acini along with sinusoidal alterations might also lead to blood supply imbalance to LL hepatocytes. Altogether, these changes could substantially limit the accessibility to blood-borne macromolecules, such as albumin-bound ICG or ^99m^Tc-mebrofenin, and therefore, LL hepatocyte functional output would be limited *in vivo*, despite a proven retained *in vitro* function, ultimately explaining *in vitro* and *in vivo* functional differences. Nevertheless, in sharp contrast, the obvious increase in the size of NLL acini could be a feasible reason behind the peculiar gain-of-function of the NLL, translating as an increased throughput capacity of bile fluids, bilirubine and other organic anions. Concerning these, the other issue needing explanation is the maintained excretion of the endogenous substrate bilirubin, whilst excretion of exogenous substrates such as ICG and ^99m^Tc-mebrofenin were temporarily diminished. While the exact hepatocyte transport of bilirubin is incompletely elucidated, it is established that bilirubin and mebrofenin (and more or less ICG) share, and thus compete for (closely) the same hepatic transport pathways^13,34,35^. Accordingly, both *in vitro* and clinical reports found reduced mebrofenin transport in hyperbilirubinemia^34,35^. For evolutionary reasons upon its toxicity, it seems plausible, that Oatps may have a higher affinity to bilirubin than to mebrofenin, ICG, or other exogenous substrates. Furthermore, increased serum levels of inflammatory cytokines, such as TNF-α and IL-6, were reported during LR^7^, leading to basolateral Oatp downregulation, thereby reducing mebrofenin uptake, whereas bilirubin may enter the cell through a different pathway or by passive diffusion^17,34^. These effects may ultimately reason different characteristics of bilirubin and mebrofenin transport. Combined, the importance of assertive clinical drug administration is underlined, for altered transport functions may lead to changes in drug pharmacokinetics and disposition, potentially evoking loss of therapeutic effect or drug intoxication.

In conclusion, our complex analyses of PVL-induced alterations in global and regional hepatic function, confirming viable and functional residual cells in the LL, substantially contributes to basic research of LR, holding future research and clinical promise regarding reversible PVO. Furthermore, the observed shift of hepatic function towards the NLL, leading to massive liver functional inhomogeneity underlines, that beyond volumetric analysis, liver functional testing is critically important during PVO procedures, for which the novel method CLE may prove useful in the future.

## Methods

### Animals and ethics

Study concept and reporting was in accordance with the Guide for the Care and Use of Laboratory Animals prepared by the National Academy of Sciences and published by the National Institutes of Health, and the 40/2013 (II.14.) Act of the Hungarian Government, as well as actual ARRIVE Guidelines^36^. The mutual approval of local, as well as institutional ethical boards (Scientific and Ethical Board of Animal Experimentation of the National Department of Food-chain Safety; the Animal Welfare Ward of the Food-chain Safety, Plant- and Land Protection Division of the Pest County Government Office; and the Animal Welfare Committee of the Semmelweis University - license numbers: PEI/001/313-4/2014; PE/EA/2895-6/2016) was also granted. Male Wistar rats (Σn = 106) weighing 210–250 g (Central Animal Facility, Semmelweis University, Budapest, Hungary) were allowed 1 week of acclimatisation and kept in an isolated, temperature- and humidity-controlled room with a 12-hour light-dark cycle and surroundings-enrichment, as well as free access to standard rat chow and water.

### Surgical procedure

Surgical procedures and analytical tests were carried out under general inhalative isoflurane anesthesia (Fortec, Cyprane Ltd., Keighley, England; 1–1.5 l/min oxygen containing 2–2.5% isoflurane (Vetflurane 1000 mg/g, Virbac, Carros, France). Rats were subjected to PVL corresponding to approximately 80% of total liver parenchyma (n = 91) according to the pre-established model of our workgroup^15,16^. After abdominal saline lavage, antibiotic [10 mg/body weight kilograms (bwkg) metronidazole intraperitoneally] and postoperative analgesic (1 mg/bwkg nalbuphine subcutaneously, repeated once 24 h later) administration, a double-layered abdominal closure was performed, and animals were rested. Other rats were spared the PVL operation to assess preoperative conditions in the three groups utilising time-point termination (n = 3 × 5) (see below).

### Experimental setting

For temporal evaluation, rats in all experimental groups were allowed different survival times declared as (preoperative) control, 24 h, 48 h, 72 h, 168 h, and 336 h, at which experimental analysis took place, with optional sacrificing subject to group allocation. Owing to different methods, five experimental groups were declared, two of which were used for ICG clearance test (n = 7), as well as for the ^99m^Tc-mebrofenin HBS and subsequent magnetic resonance imaging (MRI)-volumetry (n = 9), where the analytical pattern comprised a preoperative- and a serial of repeated postoperative scans in the above time points. Three other groups were dedicated for the acquisition of blood, bile, and tissue samples (n = 30), CLE data (n = 30), and cell cultures (n = 30). These three groups utilised individual time point termination (n = 5 each) (see Supplementary Information, Table 1).

### Conventional liver lobe analysis - weight and volume

In two separate groups, the wet weights and volumes of the LL and the NLL of the liver were determined gravimetrically (AG 245, Mettler-Toledo LLC, Columbus, OH), as well as with serial MRI-volumetry scans (nanoScan PET/MRI; Mediso Ltd., Budapest, Hungary), respectively, and expressed as a fraction of body weight (bw) (see Supplementary Information).

### Hepatocyte isolation and culturing

For *in vitro* experiments, hepatocytes were isolated by collagenase digestion as previously described^37^ with a modification of using ‘retrograde’ liver perfusion through the suprahepatic inferior vena cava allowing perfusion of both ligated and non-ligated lobes. After collecting cells separately from LL and NLL, hepatocytes were seeded onto collagen-coated plates in Williams E Medium containing a series of factors for hepatocyte culturing^38^, and kept in humidified, 5% CO_2_-enriched atmosphere at 37 °C, receiving fresh medium every 24 h. The cultures were overlaid with Matrigel after 24 h (see Supplementary Information).

### Immunofluorescence (IF) staining

Liver samples of identical anatomical loci of LL and NLL were excised, immediately frozen in liquid nitrogen, and cut by cryomicrotome. Representative cryosections of distinctive time points (control, 72 h as mid-time, and 336 h as end-stage) of LR, as well as isolated hepatocytes 72 h in culture from all time points were fixed, permeabilised, blocked, and subjected to primary antibodies against Bsep and Ntcp (both antibodies from Bruno Stieger, University Hospital Zürich, Switzerland), Mrp2 (George Scheffer, Free University Medical Center, Amsterdam, Netherlands), as well as the tight junction protein ZO-1 (Merck Millipore, Billerica, MA). After washes, the samples were stained with fluorophore-conjugated goat anti-rabbit, anti-mouse, and anti-rat secondary antibodies (Thermo Fisher, Waltham, MA), as well as with DAPI for nuclear staining. The blue, green, and red fluorescence images of stained hepatocytes and liver sections were acquired by wide-field and confocal microscopy (Leica, Wetzlar, Germany), respectively. As negative controls, samples subjected to no primary antibodies were used (see Supplementary Figure 6). Images were analysed by ImageJ 1.51 h (National Institutes of Health, Bethesda, MD) (see Supplementary Information).

### *In vitro* taurocholate- and bilirubin transport analysis

Bilirubin and taurocholate transport experiments were performed 72 h after culturing, when functional bile canaliculi were formed between adjacent cells, as described previously^38,39^. For bilirubin transport analysis, cells were provided 10 μM bilirubin in Hank’s balanced salt solution (HBSS) for 5 min, followed by washing and an efflux period in either standard or Ca^2+^/Mg^2+^-free HBSS for 10 min. Then the cells were lysed with an acetonitrile/water solution. The bilirubin and its mono- and diglucuronide conjugate content of both the efflux media and the cell-lysate were determined by high-performance liquid chromatography (HPLC). From these results, basolateral uptake, canalicular- and sinusoidal efflux transport rates, as well as intracellular accumulation of bilirubin were calculated. The experimental procedure for TC transport analysis was similar to that of the bilirubin transport, while applying ^3^H-TC as substrate at 1 μM for 1 min. The efflux lasted for 10 min, and the cells were lysed with 0.5% Triton X-100 in phosphate-buffered saline. TC content of the efflux media and the lysates were determined by liquid scintillation counting (see Supplementary Information).

### *In vivo* assessment of bile excretion

Serum and bile samples were collected in the tissue harvest group. After median laparotomy, selective biliary drainage of the LL and NLL was realised with the insertion and glue-fixation of 1.8 F polyethylene cannulas (PE10, Harvard Apparatus, Holliston, MA) into anatomically corresponding bile ducts (see Supplementary Figure 4)^15^. The amount of bile produced by the LL and NLL over 40 min was measured gravimetrically and expressed as a fraction of bw. Furthermore, citrate-anticoagulated serum samples were prepared with routine laboratory techniques from the blood acquired during exsanguination. The amounts of UCB and BG were determined by HPLC from both bile and serum samples (see Supplementary Information).

### Indocyanine-green clearance test

The ICG-clearance test was performed as described previously^15^ with the modification of ICG injected through the lateral tail vein for repeatability. Results were displayed as PDR and RT15 values (see Supplementary Information).

### Planar ^99m^Tc-mebrofenin hepatobiliary scintigraphy

After the intravenous injection of 150 MBq ^99m^Tc-mebrofenin [combination of Bromo-Biliaron (Medi-Radiopharma Ltd., Budapest, Hungary) and ^99m^Tc isotopes (Ultra-Technekow Technetium Generator, Mallinckrodt Medical, Petten, Netherlands)], planar HBS was acquired using an ultrahigh resolution collimator (NanoSPECT/CT Silver Upgrade and NanoSPECT-UHR, Mediso Ltd., Budapest, Hungary) in three dynamic phases of 20/6/2 frames per minutes for 2/4/35 minutes, respectively. By allocation of regions of interests (ROI) to the blood pool, LL, NLL, and the duodenum, tracer B_1/2_, D_START_, and the relative ratio of LL or NLL PCs to corresponding blood counts were determined (see Supplementary Information).

### Confocal laser endomicroscopy

Following laparotomy, the endoscopic wire probe of the laser unit (Cellvizio, MaunaKea Technologies, Paris, France) was motionlessly fixed to the (stabilised) inferior right lateral lobe of the NLL. After the intravenous injection of 1.5 mg/bwkg ICG in distilled water, a 40-minute time lapse video was acquired, allowing ROI allocation to liver acini and subsequent determination of T_MAX_ and ICG T_1/2_ values (see Supplementary Information).

### Statistical analysis

Data was expressed as mean ± standard deviation. For parametric data analysis of variance with Bonferroni’s *post hoc* test was performed. Pearson’s correlation test was used to correlate parametric data. *P*-values of less than 0.05 were considered statistically significant. Calculations and visualisation were performed with GraphPad Prism 6 (GraphPad Software Inc., La Jolla, CA) and Origin (OriginLab Corporation, Northampton, MA).

### Data availability

The datasets generated during and/or analysed during the current study are available from the corresponding author on reasonable request.

## Electronic supplementary material

Supplementary Information
