# Supplementary material for: Functional shift with maintained regenerative potential following portal vein ligation
Source: Sci Rep. 2017 Dec 22;7:18065. doi: 10.1038/s41598-017-18309-7 (PMC5741735; doi:10.1038/s41598-017-18309-7)
Supplement: Supplementary file 1 — Supplementary Information [file 41598_2017_18309_MOESM1_ESM.pdf]

Supplementary Information

for

Functional shift with maintained regenerative potential following portal vein ligation

Tibor Kovács[1■], Domokos Máthé[2,3■], András Fülöp[1], Katalin Jemnitz[4], Attila Bátai-Konczos[4], Zsuzsanna Veres[4]; György Török[5], Dániel Sándor Veres[2], Ildikó Horváth[2], Krisztián Szigeti [2], László Homolya[5¶], Attila Szijártó[1¶*]

■;¶: both authors contributed equally to the manuscript

*: corresponding author

[1]Hepato-Pancreatico-Biliary Surgery Research Center Hungary, 1st Department of Surgery, Semmelweis University, Budapest, Hungary

[2]Department of Biophysics and Radiation Biology, Semmelweis University, Budapest, Hungary

[3]CROmed Translational Research Centers, Budapest, Hungary

[4]Institute of Organic Chemistry, Research Centre for Natural Sciences, Hungarian Academy of Sciences, Budapest, Hungary

[5]Institute of Enzymology, Research Centre for Natural Sciences, Hungarian Academy of Sciences, Budapest, Hungary

Methods

Conventional liver lobe analysis - weight and volume

In the sample harvest group, after bile collection and exsanguination, ligated lobes (LL) and non-ligated lobes (NLL) of the liver were harvested, and their wet weights were determined gravimetrically (AG 245, Mettler-Toledo LLC, Columbus, OH; confidence: 0.01mg/0.1mg). Another group of animals were subjected to serial hepatobiliary scintigraphy (HBS) and magnetic resonance imaging (MRI) volumetry [coronal T1-weighed gradient echo sequencing, 128 axial slices of 0.4 mm thickness] (nanoScan PET/MRI; Mediso Ltd., Budapest, Hungary). *In vivo* liver lobe volumes were determined following manual delineation of LL and NLL on each axial slice and three-dimensional reconstruction in a 160x160 matrix. Both parameters (weight and volume) were ultimately expressed as a fraction of body weight (bw).

Hepatocyte isolation and generation of cell cultures

For hepatocyte isolation, a three-step retrograde perfusion procedure was applied, since the portal vein branches of the median-, left lateral- and caudate lobes had been ligated. A glass cannula was inserted into the suprahepatic inferior vena cava, and the liver was first flushed with 300 ml of Ca^{2+}-free Earle's balanced salt solution (EBSS) (self-produced) containing EGTA. The liver was then perfused with the same buffer without the chelating agent and finally with EBSS containing Ca^{2+} and type IV collagenase (prepared from *Clostridium histolyticum*, Sigma-Aldrich, St. Louis, MO) for approximately 5 min. The portal vein was incised to allow efflux. The flow rate was 30 ml/min and all perfusion solutions were pre-oxygenised and temperature-controlled at 37°C. Perfusion was considered successful when the whole organ was completely blanched and the endpoint when the tissue was visibly digested and the capsule started to separate from the liver surface after approximately 5 mins.

Following the precise separation of the LL and the NLL, cell viability was determined by trypan blue exclusion. From the separate suspensions of the LL and the NLL, only hepatocytes from preparations with more than 90 % viability were plated at a density of 2.0 x 10^6 cells/well on 6-well plates, and 0.36 x 10^6 cells/well on 24-well plates, in William's Medium E containing 5% of fetal calf serum, 0.1μM insulin, 0.05 μM glucagon, 0.05 mg/ml gentamicin, 30 nM Na_2SeO_3, and 0.1μM dexamethasone. Calf serum was present for the first 24h and then omitted. Cells were maintained at 37°C in a humidified atmosphere of 95% air and 5% CO_2. Twenty-four hours after plating, cells were overlaid with matrigel basement membrane matrix (Matrigel Matrix; SoftFlow Hungary, Pecs, Hungary) at a concentration of 0.25 mg/ml in 2 ml of ice-cold William's Medium E supplemented with insulin, glucagon, gentamicin, dexamethasone, and Na_2SeO_3 to achieve sandwich configuration. The culture medium was replaced every 24h.

In vitro Ntcp, Bsep and ZO-1 immunofluorescence staining

For immunofluorescence (IF) staining of isolated hepatocytes, the cells 72h in culture were fixed and permeabilised with 4% paraformaldehyde and 0.1% Triton X-100 in phosphate buffered saline (PBS) for 1h, then with ice cold methanol for an additional 15 min, and blocked in the blocking buffer of Dulbecco's modified PBS (DPBS) containing 2 mg/ml bovine serum albumin (BSA), 1 % fish gelatin, 0.1 % Triton X-100, and 5 % goat serum (pH 7.2). For staining of sodium-taurocholate cotransporting polypeptide (Ntcp), bile salt export pump (Bsep) and tight junction protein zonula occludens-1 (ZO-1), rabbit K44 (1:200), rabbit K4 (1:250) (both antibodies obtained from Bruno Stieger, University Hospital Zürich, Zürich, Switzerland), and rat MABT11 (1:200, Merck Millipore, Billerica, MA) antibodies were used, respectively. After washing, the samples were stained with Alexa Fluor 488-conjugated or Alexa Fluor 594-conjugated goat anti-rabbit IgG H&L (1:250) along with Alexa Fluor 488-conjugated goat anti-rat IgG H&L (1:250) secondary antibodies (Thermo Fisher, Waltham,

MA). Finally, the nuclei were stained with 1 μM 4',6-diamidino-2-phenylindole (DAPI) in DPBS for 10 min. The blue, green, and red fluorescence of stained samples were visualised by a Leica SP8 confocal laser scanning microscope using a HC PL APO CS2 40× (NA=1.25) oil immersion objective lens (Leica, Wetzlar, Germany) at 405, 488, and 552 nm excitations, respectively. Samples subjected to the same fixation, permeabilization, blocking, and staining procedures with the exception of incubation with the primary antibodies were used as negative controls for the staining (Supplementary Figure 6). For visualisation ImageJ 1.51h software (National Institutes of Health, Bethesda, MD) was used.

In vitro bilirubin transport experiments

Bilirubin transport experiments were performed 72h after culturing as described previously. [1] Briefly, cells were incubated with 10 μM bilirubin for 5 min in standard Hanks' Balanced Salt Solution (HBSS) at 37°C. After washing off the uptake medium, the cells were incubated with either standard or Ca^{2+}/Mg^{2+} free HBSS containing 1 mM EGTA. Efflux was allowed for 10 min, after which the cells were lysed with an acetonitrile/water solution [30% (v/v)]. The amounts of bilirubin and its mono- and diglucuronide conjugates (BMG, BDG) in the efflux medium and in the cell lysates were analysed by high-performance liquid chromatography (HPLC). In standard HBSS, the bile canalicular networks maintain their integrity, whereas in calcium-free HBSS the tight junctions are disrupted and the content of the canaliculi leaks into the efflux medium. The biliary transport was determined by subtracting the amount of bilirubin and its conjugates in standard efflux medium from that in Ca^{2+}/Mg^{2+} free efflux medium, and was expressed as nmol/mg protein. Sinusoidal transport was determined in the standard efflux medium. The intracellular accumulation was measured in the lysate of cells incubated in Ca^{2+}/Mg^{2+} free medium. Assays were run using three wells as one set from at least three cell preparations. The HPLC method for analysing bilirubin and conjugated bilirubin (BG) was performed as described previously [1].

In vitro taurocholate transport experiments

Taurocholate (TC) uptake experiments were performed 24h after plating, as described previously. [2] Briefly, the wells were washed once with HBSS. The uptake experiment was started by the addition of HBSS containing 1 μM ^{3}H-TC and lasted for 1 min at 37°C. The uptake was terminated by the removal of the substrate-containing buffer, and the wells were washed three times with ice-cold HBSS. Then the cells were lysed with 0.5% Triton X-100 solution. The intracellular radioactivity was determined by liquid scintillation counting. Assays were run using four wells in one set; all experiments were carried out with hepatocytes from three independent cell preparations. TC efflux experiments were performed similarly to that of with bilirubin transport 72h after plating. ^{3}H-TC was applied at 1 μM. The uptake period was 1 min and the efflux lasted for 10 min. Following the efflux period, the cells were lysed with 0.5 % Triton X-100 in PBS. The amount of TC in the efflux medium and the cell lysates were determined by liquid scintillation counting. Assays were run using three wells as one set. All experiments were carried out with cultures from at least three independent cell preparations. Data were normalised for the protein content analysed in wells where the cells were incubated with standard HBSS (Pierce BCA Protein Kit, Thermo Scientific).

In vivo bilirubin transport experiments

The amount of bilirubin, and BG metabolites [BMG: bilirubine monoglucuronide; BDG: bilirubine diglucuronide] in the bile and serum samples, collected as described in the article, was determined by HPLC with the same method as in case of the *in vitro* experiments. The bile samples were diluted hundredfold with distilled water, and the protein content of the serum was precipitated with 3x volume of acetonitrile before injection.

In vivo Ntcp and Mrp2 immunofluorescence staining

For *in vivo* immunohistochemistry, liver samples of identical anatomical loci of the LL and NLL of control, 72h and 336h survivors were excised after liver weight measurements, and immediately frozen in liquid nitrogen. Tissue sections were cut by cryomicrotome, fixed in pre-chilled methanol (-20°C) for 10 min, and blocked with the same blocking buffer as described with in vitro IF. The samples were subjected to K4 rabbit anti-Ntcp antibody (1:200, obtained from Bruno Stieger) and M_2III-6 mouse anti-Mrp2 antibody (1:20, obtained from George Scheffer, Free University Medical Center, Amsterdam, Netherlands) for 1h, washed, and stained with Alexa Fluor 488-conjugated goat anti-rabbit IgG H&L (1:250) and Alexa Fluor 594-conjugated goat anti-mouse IgG H&L (1:250) secondary antibodies (Thermo Fisher, Waltham, MA). Samples were visualised by a Leica SP8 confocal laser scanning microscope using a HC PL APO CS2 40× (NA=1.25) oil immersion objective lens (Leica, Wetzlar, Germany) at 488 and 594 nm excitations, respectively. As negative controls, samples subjected to the procedures with the exception of incubation with the primary antibodies were used (Supplementary Figure 6). For visualisation ImageJ 1.51h software (National Institutes of Health, Bethesda, MD) was used.

Indocyanine-green clearance test

The indocyanine-green (ICG)-clearance test was performed similarly to the previous study of our workgroup [3]. The medial side of the left upper thigh was shaved, and a neonatal laser probe (PV50200 disposable sensor for neonates; PULSION Medical Systems, Feldkirchen, Germany) of a commercially available analysing device (PC5000 LiMON; PULSION Medical Systems) for ICG densitometry was fixed with an elastic bandage. Following automatic calibration and test initiation, 1 ml/bwkg of 1.5 mg ICG/ml distilled water was injected into the lateral tail vein. Results were displayed 5-6 minutes later as plasma disappearance rate (PDR) and 15-minute retention (RT15) values.

Planar ^{99m}Tc-mebrofenin hepatobiliary scintigraphy

^{99m}Tc-mebrofenin [^{99m}Tc-2,4,6 trimethyl-3-bromo iminodiacetic acid], produced by combining Bromo-Biliaron radiopharmaceutical kit (Medi-Radiopharma Ltd., Budapest, Hungary) and ^{99m}Tc isotopes, generated with an Ultra-Technekow Technetium Generator (Mallinckrodt Medical, Petten, The Netherlands), was injected in 150 MBq dosage in 0.3 ml saline into the tail vein. Thereafter, planar HBS (NanoSPECT/CT Silver Upgrade, Mediso Ltd., Budapest, Hungary) was acquired as projections from four separate angles in a resolution of 256 x 256 using Ultrahigh Resolution (UHR) parallel septal collimator (NanoSPECT-UHR, Mediso Ltd, Budapest, Hungary). A dynamic protocol of three different phases was used including 20/6/2 frames per minutes for 2/4/35 minutes, respectively, to monitor the rapid uptake and the canalicular elimination of the tracer. Recordings were evaluated with manual allocation of elliptic regions of interest (ROI) to the anteroposterior projection corresponding to the blood pool, as well as the LL and NLL. With respect to its more elusive position, the duodenal ROI was placed considering projections from all four angles. The characteristic parameters derived from the kinetics curves included the blood half-life ($B_{1/2}$), first duodenal appearance (D_{START}), as well as regional-specific information of LL and NLL – time of maximum, tracer half-life, and relative ratio of LL or NLL peak counts (PC) to corresponding blood counts.

Confocal laser endomicroscopy

Following laparotomy, the inferior right lateral lobe of the liver was mobilised, and carefully placed on a fastened plastic foil, elevated to the level of about the median axillary plane while preserving portal inflow, to attenuate respiration-associated movement. The endoscopic wire probe of the laser unit (Cellvizio, MaunaKea Technologies, Paris, France) was harmlessly pressed against and stabilised on the medial surface of the inferior right lateral lobe. After an injection of ICG (1 ml/bwkg of 1.5 mg ICG/ml distilled water) into the tail vein, a 40-minute time lapse was acquired. Video evaluation was performed by manual ROI allocation to liver

acini with the exclusion of large vessels. Exponential growth and decay were used to determine time of signal maximum (T_{MAX}) and ICG half-life ($T_{1/2}$) values, respectively.

References

1 Lengyel, G., Veres, Z., Szabó, P., Vereczkey, L. & Jemnitz, K. Canalicular and sinusoidal disposition of bilirubin mono-and diglucuronides in sandwich-cultured human and rat primary hepatocytes. *Drug metabolism and disposition* **33**, 1355-1360 (2005).

2 Jemnitz, K., Veres, Z. & Vereczkey, L. Contribution of high basolateral bile salt efflux to the lack of hepatotoxicity in rat in response to drugs inducing cholestasis in human. *Toxicological sciences* **115**, 80-88 (2010).

3 Fulop, A. *et al.* Alterations in hepatic lobar function in regenerating rat liver. *The Journal of surgical research* **197**, 307-317, doi:10.1016/j.jss.2015.04.033 (2015).

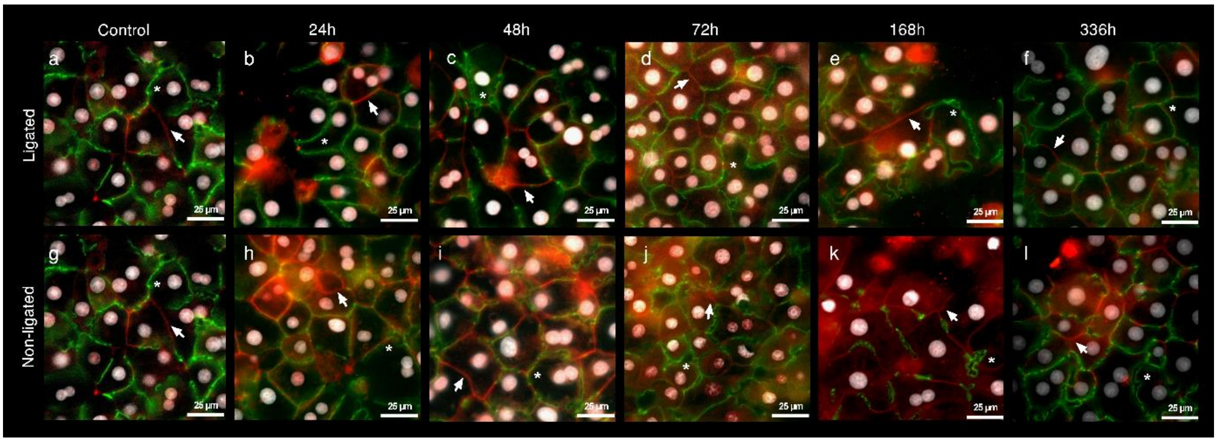

Supplementary Figure 1. ***In vitro*** **Ntcp and ZO-1 immunofluorescence.** A series of images demonstrating the expression and localisation of the sodium-taurocholate cotransporting polypeptide (Ntcp) [labelled as red] and zonula occludens-1 (ZO-1) [labelled as green] in cultured hepatocytes isolated from the ligated- ***(a-f)*** and non-ligated ***(g-l)*** liver lobes of rats at the indicated time points after portal vein ligation (PVL). The cells were kept in cultures for 72h prior to immunofluorescence staining. Hepatocytes, regardless of lobar or temporal origin, maintained their ability of generating viable cultures, forming bile canaliculi correctly sealed with ZO-1, and properly expressing Ntcp. Cell nuclei stained with DAPI are shown in grey. Representative markings indicate ZO-1-positive intercellular connections (asterisks), as well as the basolateral expression of Ntcp (arrowheads). White bars represent 25 µm.

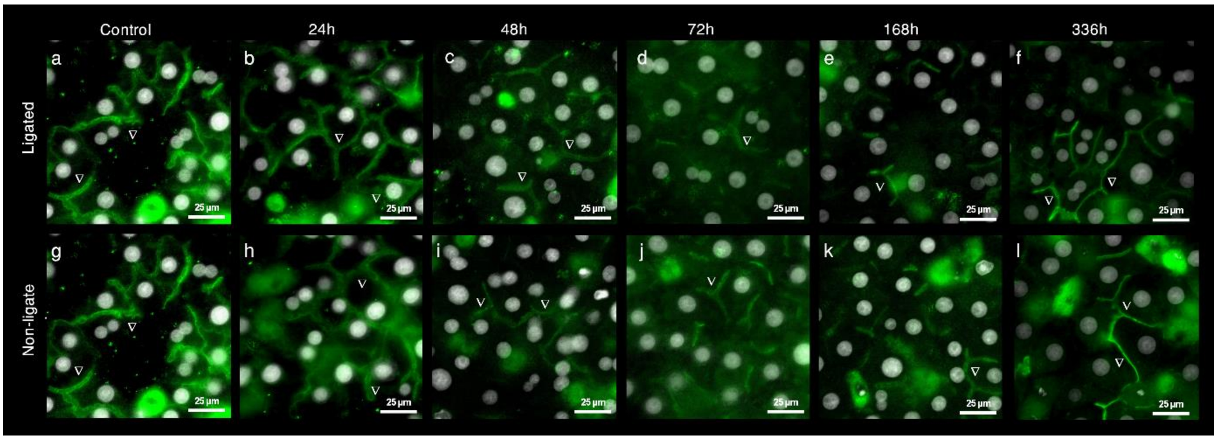

Supplementary Figure 2. ***In vitro*** **Bsep immunofluorescence.** A series of images demonstrating the expression and localisation of the bile salt export pump (Bsep) [labelled as green] in hepatocytes 72h in cultures isolated from the ligated- ***(a-f)*** and non-ligated ***(g-l)*** liver lobes at the indicated time points after portal vein ligation (PVL). Cells, regardless of lobar or temporal origin, maintained their ability of generating viable cultures with proper bile canaliculi, and expressing Bsep in the canalicular membrane. Cell nuclei stained with DAPI are shown in grey. Representative markings (triangles) indicate specific staining of bile canaliculi. White bars represent 25 µm.

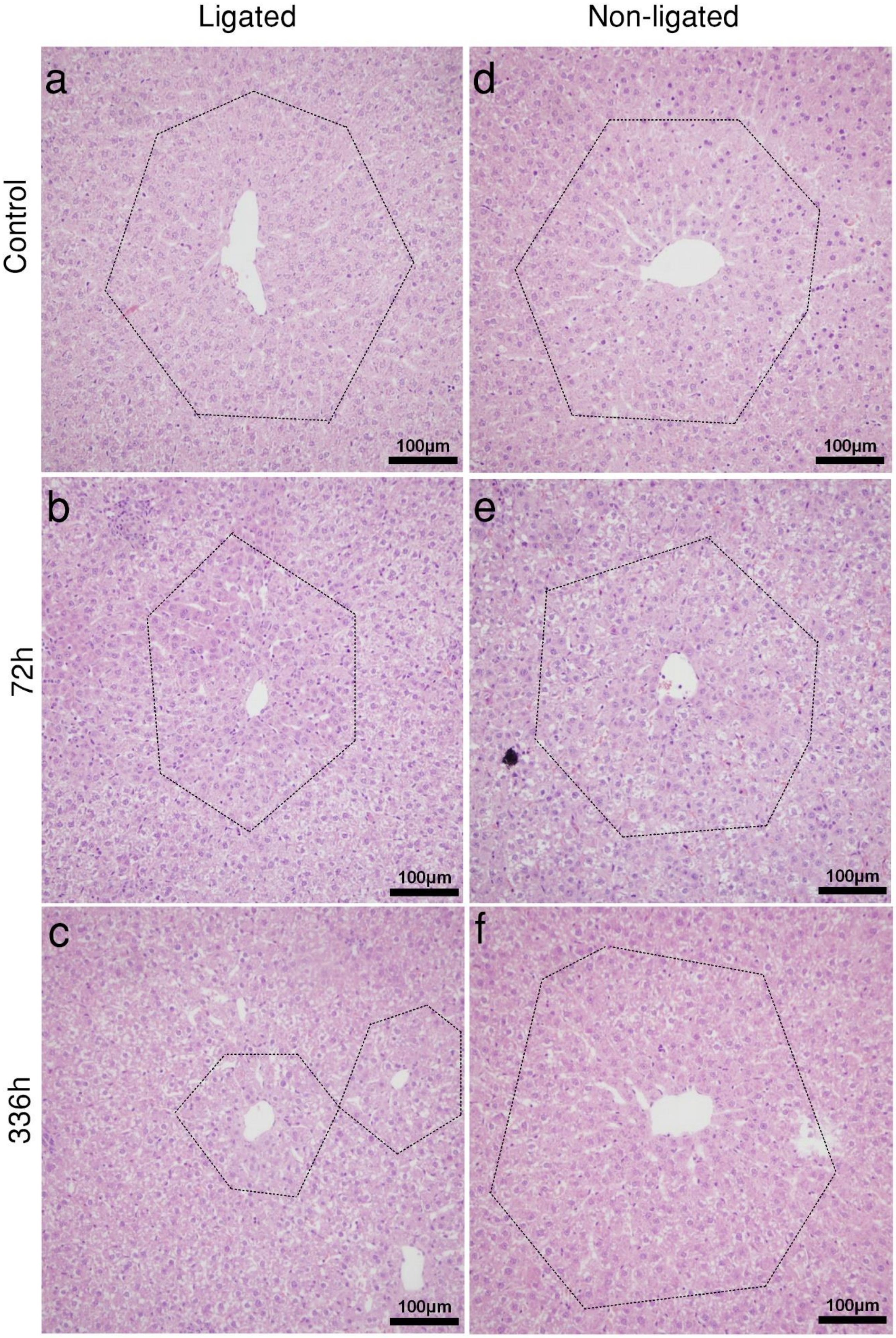

Ligated
Non-ligated
Control
72h
336h
a
d
b
e
c
f
100μm

Supplementary Figure 3. Histopathology with H-E staining. Representative pictures of histopathological slides of hematoxylin-eosin (H-E) stained sections from liver specimens harvested from identical anatomical loci of ligated (LL) ***(a-c)*** and non-ligated (NLL) liver lobes ***(d-f)*** of control rats, as well as animals 72h and 336h after portal vein ligation (PVL). While control conditions exhibiting physiological liver histology were matching ***(a, d)***, in parallel with the progressive development of atrophy and hypertrophy, sections of the LL and NLL at 72h and 336h after PVL were markedly different. At 72h, LL sections showed mainly pericentral patches of increased cytoplasmic eosinophilia and blurred cell borders, as well as predominantly periportal infiltration of leukocytes which together are likely representations of healing necroapoptotic lesions and a subsequent increase in cell inflammatory activation to clean dead cells and debris. Sinusoidal dilation, owing to the hemodynamic consequences of increased arterial influx following portal vein ligation, was also notable ***(B)***. On 72h sections of the NLL, these characteristics were absent, meanwhile, cells were enlarged and showing signs of mildly increased fat content ***(e)***. 336h sections showed the consolidation of (likely ceasing) histopathological alterations, by which LL slides exhibited significantly shrunk cells and liver acini, with no signs of necroapoptotic patches ***(c)***. Lesions or necoapoptotic patches were also absent from 336h NLL sections, whereas liver acini were notably enlarged ***(f)***. Summarizing, the course and characteristics of histopathological alterations pertaining to presence of necroapoptotic lesions and cellular- and liver acini size were diverging between LL and NLL. Observed changes are in accordance with previously described histological alterations following PVL. Furthermore, these are in great corroboration with the current observations of immunofluorescence staining of *ex vivo* liver specimens for sodium-taurocholate cotransporting polypeptide (Ntcp) and canalicular multispecific organic transporter (Mrp2) immunofluorescence (see Figure 4) showing the same pattern of cellular and liver acini size changes, and similar characteristics of necroapoptotic lesions in the LL at

72h, supported by absence of respective transporters. Dashed line markings represent arbitrary acinar borders. Black bars represent 100 μm.

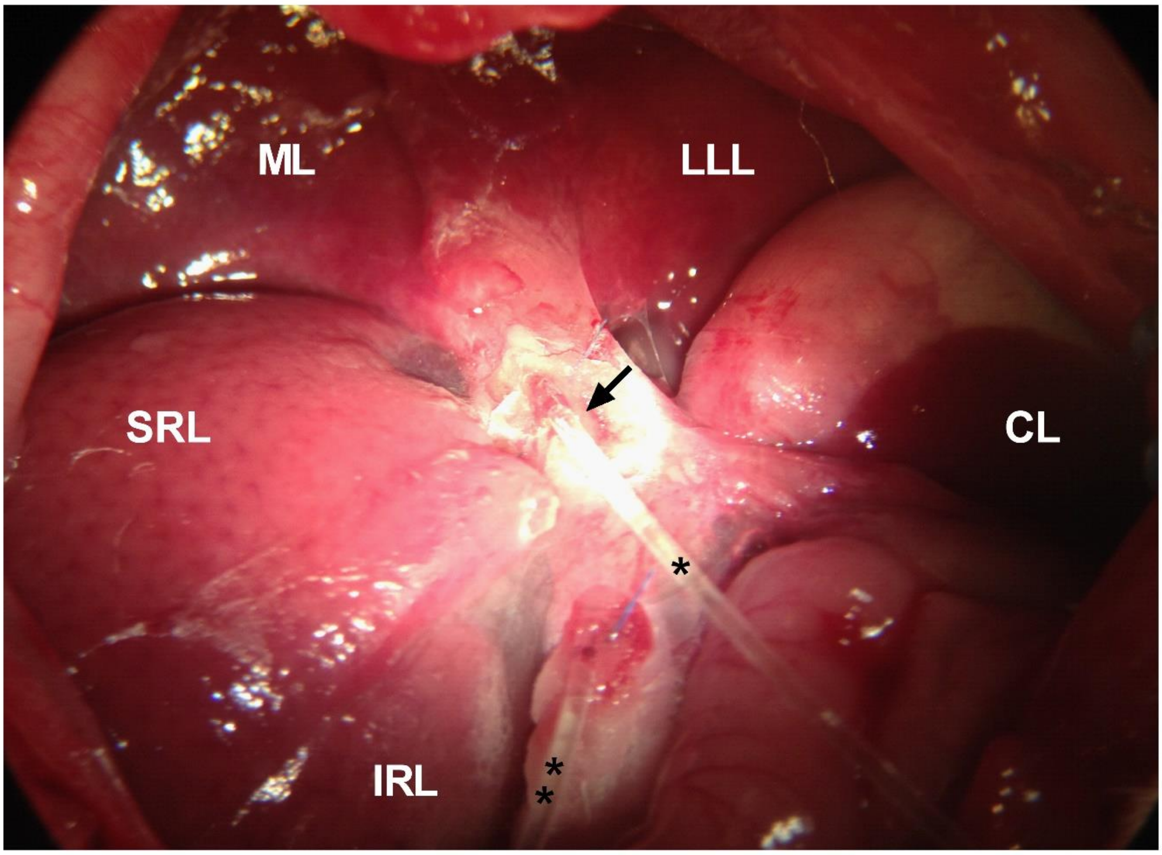

Supplementary Figure 4. Selective biliary drainage. To isolate the bile effluent of ligated and non-ligated lobes, the common bile duct of the median- (ML), left lateral (LLL) and caudate (CL) liver lobes was ligated (marked with arrowhead) just above the debranching of the common bile duct of the superior right lateral (SRL) and inferior right lateral (IRL) lobes. Next, a 1.8F polyethylene cannula (marked as ‘*’) was inserted and glue-fixed into the common bile duct of the ML, LLL, and CL, as well as another one (marked as ‘**’) into the *ductus choledochus,* containing the bile effluent of the SRL and IRL, thereby allowing the selective collection of biliary samples for 40 min from ligated and non-ligated lobes, respectively.

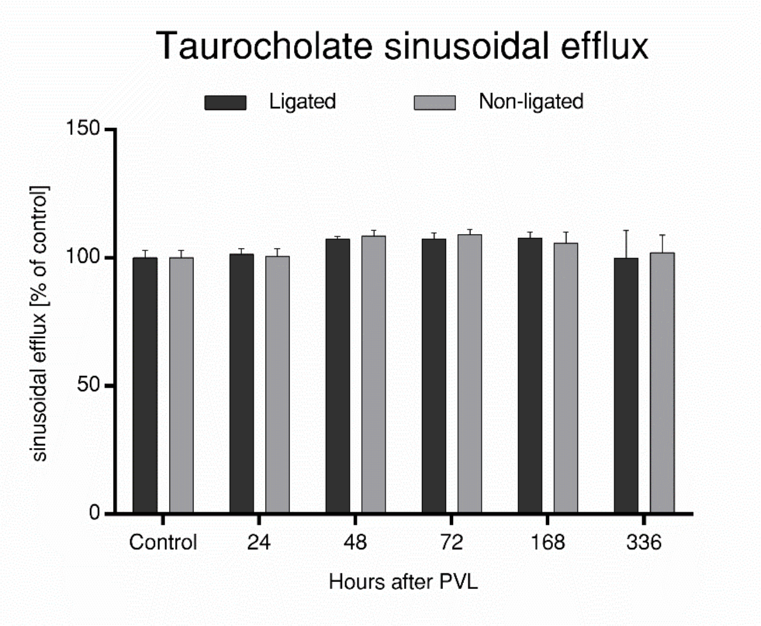

Supplementary Figure 5. ***In vitro*** **taurocholate sinusoidal efflux.** The specific taurocholate transport of hepatocytes 72h in cultures prepared from ligated- and non-ligated lobes at the indicated time points after portal vein ligation (PVL) was measured *in vitro*. As part of the described hypothesised adaptive response, the sinusoidal efflux of taurocholate was temporarily increased between 48 and 168h in both lobes; however, values remained below the level of statistical significance. Statistical analysis was performed with analysis of variance (ANOVA) with Bonferroni's *post hoc* test to correct for multiple comparisons. Results are given as means ± standard deviation, with n=5 animals per time points. For additional information, see the text and the legends for Figure 3.

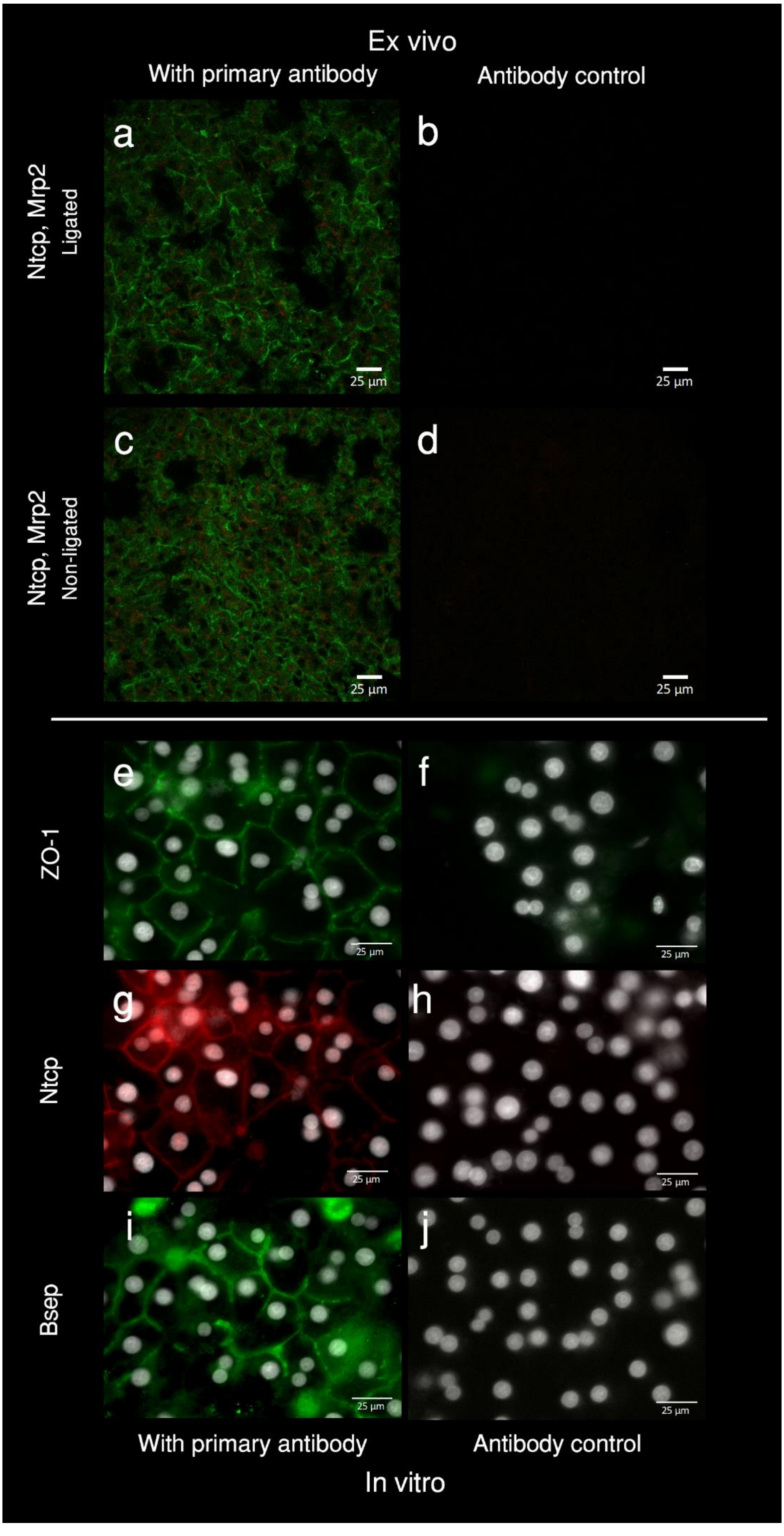
Ex vivo
With primary antibody
Antibody control
Ntcp, Mrp2
Ligated
Ntcp, Mrp2
Non-ligated
a
b
c
d
25 µm
ZO-1
Ntcp
Bsep
e
f
g
h
i
j
With primary antibody
Antibody control
In vitro

Supplementary Figure 6. Control staining for immunofluorescence labelling. Immunofluorescence staining for sodium-taurocholate cotransporting polypeptide (Ntcp) [labelled as green] and canalicular multispecific organic transporter (Mrp2) [labelled as red] were performed on sections of snap frozen liver specimens excised from ligated lobes ***(a)*** and non-ligated lobes ***(c)*** as shown on Figure 4. In parallel, the expression and localisation of zonula occludens-1 (ZO-1) [labelled as green] ***(e)***, Ntcp [labelled as red] ***(g)***, as well as bile salt export pump (Bsep) [labelled as green] ***(i)*** was also checked in hepatocytes 72h in cultures isolated from the ligated liver lobes, as shown on Figure 2 and Supplementary Figures 1 and 2. The background fluorescence of the both liver sections ***(b, d)*** and cultured hepatocytes ***(f, h, j)*** was determined by samples subjected to the same procedures but omitting the primary antibodies during staining. Similar low background fluorescence was observed in hepatocytes isolated form the non-ligated lobes (not shown). For the *in vitro* cultures, the nuclei were stained with DAPI shown in grey. White bars represent 25 μm.

Time points of analysis: control/24h/48h/72h//168h/336h		
**Group**	**Method**	**Analytical pattern**
**Mebrofenin** (n=9)	planar ^{99m}Tc-mebrofenin HBS, MRI	serial tests
**ICG** (n=7)	ICG-clearance test	serial tests
**Tissue harvest** (n=30)	bile, serum, liver sample harvest	time-point termination
**Cell culture** (n=30)	cell culture IF and *in vitro* transport analysis	time-point termination
**CLE** (n=30)	confocal laser endomicroscopy	time-point termination

Supplementary Table 1. Summary of experimental settings and analytical methods. The experimental setup included n=106 of 210-250g male Wistar rats in a total of five experimental groups, for a range of analytical methods in either a serial testing-, or time-point termination pattern. Time points of analysis were declared as (preoperative) control, and 24h/48h/72h/168h/336h after portal vein ligation. The 'Mebrofenin' and 'ICG' groups utilised serial functional testing including planar ^{99m}Tc-mebrofenin hepatobiliary scintigraphy (HBS) and magnetic resonance imaging (MRI), as well as the indocyanine-green (ICG) clearance test. The other three groups incorporated time-point termination for the harvest of tissue (bile, serum, and liver) samples, generation of cell cultures for *in vitro* immunofluorescence (IF) and transport analysis, as well as segmental liver functional measurement with confocal laser endomicroscopy (CLE).
